# B Cells with Regulatory Function in Animal Models of Autoimmune and Non-Autoimmune Diseases

**DOI:** 10.4236/oji.2015.51002

**Published:** 2015-02-12

**Authors:** Mei Lin, Zuomin Wang, Xiaozhe Han

**Affiliations:** 1 Department of Immunology and Infectious Diseases, The Forsyth Institute, Cambridge, USA; 2 Department of Stomatology, Beijing Chao-Yang Hospital, Capital Medical University, Beijing, China

**Keywords:** Bregs, Animal Models, IL-10, Autoimmune Disease, Immune Regulation

## Abstract

Although the identification of B cell subsets with negative regulatory functions and the definition of their mechanisms of action are recent events, the important negative regulatory roles of B cells in immune responses are now broadly recognized. There is an emerging appreciation for the pivotal role played by B cells in several areas of human diseases including autoimmune diseases and non-autoimmune diseases such as parasite infections and cancer. The recent research advancement of regulatory B cells in human disease coincides with the vastly accelerated pace of research on the bridging of innate and adaptive immune system. Current study and our continued research may provide better understanding of the mechanisms that promote regulatory B10 cell function to counteract exaggerated immune activation in autoimmune as well as non-autoimmune conditions. This review is focused on the current knowledge of BREG functions studied in animal models of autoimmune and non-autoimmune diseases.

## 1. Introduction

Historically, B cells have been characterized as positive regulators of humoral immune responses and are distinguished by their ability to terminally differentiate into antibody (Ab)-secreting plasma cells [1] [2] or serve as antigen (Ag)-presenting cells (APCs), for optimal Ag-specific CD4^+^ T-cell expansion, memory formation, cytokine production [3]–[5] and positively regulate CD8^+^ T-cell responses by expression of co-stimulatory molecules [6]–[8].

Evidence for B-cell negative regulatory function has accumulated over the past 30 years. The hypothesis that suppressor or regulatory B (Bregs) cells orchestrate the immune system was originally proposed in the 1970s and maintains that the suppressive function of B cells was mainly restricted to their ability to produce “inhibitory” antibodies [9]. These initial findings were later followed by a flurry of seminal papers supporting a “suppressive framework” for B cells and a link with T-cell tolerance [10]–[13]. Later studies showed that a B-cell-restricted IL-10 deficiency had a similar exacerbating effect on EAE [14] [15] and rheumatoid arthritis [16], suggesting that activated B cells exerted regulatory activity that resolved the inflammation. From this, this term “regulatory B cell” was coined.

More recently, a relatively rare negative regulatory B-cell subset was identified that was predominantly contained within a phenotypically unique CD1d^hi^CD5^+^CD19^hi^ subset in the spleens of naive wild-type mice [17]. This regulatory B-cell subset is Ag-specific and significantly influences T-cell activation and inflammatory responses through IL-10 production [17] [18]. Given that multiple regulatory B cell subsets are likely to exist, as now recognized for T cells, it has been specifically labeled this IL-10 competent CD1d^hi^CD5^+^CD19^hi^ regulatory subset as B10 cells because they are responsible for most IL-10 production by B cells and they appear to only produce IL-10 [19].

Recently, many studies have used animal models of human diseases to demonstrate that B cells have regulatory functions *in vivo*, and most of them use B-cell-deficient μMT mice or the adoptive transfer of bulk B cells. In addition, multiple groups have identified IL-10-producing regulatory B cells of varying phenotypes in different disease models following diverse stimulation and culture protocols [14]–[16] [20]–[27].

## 2. Bregs in Autoimmune Diseases

### 2.1. Experimental Autoimmune Encephalomyelitis

B cells play a dual role in experimental autoimmune encephalomyelitis (EAE), which is the animal model of the human autoimmune disease multiple sclerosis (MS). The first one is contributing to the pathogenesis of EAE through the production of anti-myelin antibodies that contribute to demyelination. The other one is playing an essential role in the spontaneous recovery from EAE.

In 1996, Wolf found that B cells were not required for the onset of EAE, revealed that μMT mice failed to spontaneously recover from EAE [28]. This was the first indication in animal autoimmune models that B cells play a regulatory role in down-regulating inflammation. While in the later study, C57BL/6μMT mice were either not able to recover from EAE when an EAE induction protocol that allowed for recovery from EAE was used, which indicating that B cell production of IL-10 and expression of CD40 were requirements for their regulatory activity [14].

In regards to TLR signaling TLR2/4 ligands are present in the CFA adjuvant not only used to induce EAE but also induce the production of IL-10 by B cells [29]. Thereby, IL-10-producing regulatory B cells, most likely B10 cells, are important for controlling EAE severity and resolution. More specifically, Myd88 expression by B cells was required for recovery from EAE in MOG-peptide EAE [29].

The Tedder laboratory studied that both genetic disruption of CD19 and the transfer of CD19^−/−^ B cells from MOG peptide-primed mice exacerbated EAE which demonstrated that signaling was important for regulatory B cell functions [30]. They analysed CD19^−/−^ and human CD19 transgenic (hCD19tg) mice, with the later harboring hyperactive B cells, indicating that CD19^−/−^ mice exhibit enhanced T cell-mediated inflammation (contact hypersensitivity (CHS)), and inflammation in hCD19tg mice was reduced [17].

The B cell regulatory effects were recently shown not to be IL-10 dependent [31]. On the contrary, the Tedder laboratory showed that the adoptive transfer of CD1d^hi^CD5^+^B cells from CD19^−/−^ mice or from MOG-sensitized animals prior to EAE induction by MOG peptide attenuated EAE disease severity. Meanwhile, B cells from IL-10^−/−^ mice did not reduce disease severity, indicating an IL-10-dependent mechanism [1]. B cell depletion therapy in early MS clinical trials with anti-CD20 showed remarkable efficacy in preventing disease progression [32] [33]. However, much still needs to be learned regarding how each B cell function can harnessed to either prevent or induce the recovery from MS.

### 2.2. Type 1 Diabetes

Studies on B10 cells and mouse models of diabetes are limited to the nonobese diabetic (NOD) mouse, a spontaneous model of type 1 diabetes in which autoimmune destruction of the insulin-producing pancreatic *β* cells is primarily T cell mediated [34].

Although B cells play the pathogenic role in T1D initiation [35], B cells activated *in vitro* can maintain tolerance and transfer protection from T1D in NOD mice, both delay the onset and reduces the incidence of T1D. Protection from T1D is IL-10 dependent since the transfusion of activated NOD-IL-10^−/−^ B cells does not confer protection from T1D or the severe insulitis observed in NOD recipients [36] [37]. In another study, LPS-activated B cells were transferred into prediabetic NOD mice and found that Fas ligand and secreted transforming growth factor-*β* were upregulated, which were considered to contribute to inhibit autoimmunity [37].

Although the animal studies in TID have shed some light on the limitation of the rarity of circulating B10 cells, the possibility of therapeutic transfusion of autologous, IL-10-producing, BCR-activated B cells or B10 cells in order to protect human subjects at risk for T1D remains elusive.

### 2.3. Arthritis

CIA is a model for human rheumatoid arthritis that develops in susceptible mouse strains immunized with heterologous type II collagen emulsified in complete Freund’s adjuvant [38] [39], which shares in common with rheumatoid arthritis having an association with a limited number of MHC-II haplotypes that determine disease susceptibility [40] [41].

B cells are important for initiating inflammation and arthritis [42]. By contrast, IL-10-producing B-cell sub-sets regulate inflammation during CIA. Activation of arthritogenic splenocytes with Ag and agonistic anti-CD40 mAb induces a B cell population that produces high levels of IL-10 and low levels of IFN*γ* [16]. Specifically, multiple studies have tested whether the adoptive transfer of activated B cells could inhibit CIA. Mauri’s lab injected CD40 mAb and collagen-activated B cells from the spleens of arthritogenic mice into recipient mice, observed that arthritis incidence (>50% reduction), disease severity (>90%), and Th1 cell differentiation are inhibited. Moreover, adoptive transfer of B cells also partially inhibits arthritis incidence and severity, even after disease initiation. However, the adoptive transfer of IL-10^−/−^ B cells does not prevent arthritis in this model system [16]. Evans has tested the adoptive transfer of B cells into mice immunized with bovine collagen (type II collagen) inhibits TH1 responses, prevents arthritis development, and is effective in ameliorating established disease, while the adoptive transfer of CD21^hi^CD23^+^IgM^+^ B cells from DBA/1 mice in the remission phase could prevents CIA and reduces disease severity through IL-10 secretion [22]; Gu also found a substantial reduction in the number of T_H17_ cells [43]. Other studies administered apoptotic thymocytes to mice up to 1 month before the clinical onset of CIA is also protective for severe joint inflammation and bone destruction [23].

Collectively, activated spleen B cells responded directly to apoptotic cell treatment, increasing secretion of IL-10, which is important for inducing T-cell-derived IL-10. Moreover, the passive transfer of B cells from apoptotic cell-treated mice provided significant protection from arthritis.

### 2.4. Systemic Lupus Erythematosus

Studies in the NZB/W spontaneous lupus model therefore suggest that B10 cells have protective and potentially therapeutic effects. In wild type NZB/W mice, the CD1d^hi^CD5^+^B220^+^ B cell subset, which is enriched in B10 cells, is increased 2.5-fold during the disease course, whereas CD19^−/−^ NZB/W mice lack this CD1d^hi^CD5^+^ regulatory B cell subset [44]. Mature B cell depletion initiated in NZB/W F1 mice, hastens disease onset, which parallels depletion of B10 cells, suggesting that B cell-negative regulatory effects are important in NZB/W mice [45]. Moreover, the potential therapeutic effect of B10 cells in lupus is highlighted by the prolonged survival of CD19^−/−^ NZB/W recipients following the adoptive transfer of splenic CD1d^hi^CD5^+^ B cells from wildtype NZB/W mice [44]. However, in the MRL.Fas(lpr) mouse lupus model, B cell-derived IL-10 does not regulate spontaneous autoimmunity [46]. The study suggests fundamental differences in the pathogenesis and immune dysregulation in the NZB/W lupus model compared with the MRL.Fas(lpr) model.

### 2.5. Inflammatory Bowel Disease

Early studies showed that B cells and their autoantibody products suppress colitis in T cell receptor alpha chain-deficient mice that spontaneously develop chronic colitis, while B cells are not required for disease initiation [47].

Mizoguchi’s group has subsequently demonstrated that B cells are the regulatory mediators and were the first to identify a B-cell subset with up regulated CD1d expression that is induced in the gut-associated lymphoid tissues of mice with intestinal inflammation, and they also suggested IL-10-producing B-cell subsets with varying phenotypes and origins regulate intestinal inflammation during inflammatory bowel disease [15] [48].

Dextran sulfate sodium-induced intestinal injury is more severe in CD19^−/−^ mice where B10 cells are absent than in wild type mice [49], suggesting these inflammatory responses are negatively regulated by CD1d^hi^CD5^+^ B cells producing IL-10. Moreover the other study observed the adoptive transfer of mesenteric lymph node B cells also suppresses inflammatory bowel disease through a mechanism that correlates with an increase in regulatory T-cell subsets [50]. Thus, cytokine-producing B cells can regulate immune-mediated gut inflammation. B10 cells therefore emerge during chronic inflammation in mouse models of inflammatory bowel disease, where they suppress the progression of inflammatory responses and ameliorate disease manifestations.

## 3. Bregs in Parasitic Infection

The first clue for an anti-inflammatory role of Bregs in infections with parasites came from a study demonstrating a regulatory role of B cells during *Schistosoma mansoni* worm plus egg infection. Infection of μMT mice led to an enlargement of hepatic granuloma and a decreased lifespan [51] [52]. Recently, it has been demonstrated that B cells induced by *Schistosoma mansoni* worms is in an IL-10-dependent manner, which are responsible for protecting mice against fatal, experimentally induced anaphylaxis [53]. Breg cell function during different stages of natural *S. mansoni* infections and showed the existence of active regulatory mechanisms during chronic, but not acute infection [54]. A similar liver pathology was observed in schistosome-infected, Fcg-chain receptor knockout mice [52], suggesting that B-cell regulation is mediated either by the production of antibodies neutralizing egg-derived inflammatory molecules or by triggering the production of anti-inflammatory mediators from FcR^+^ cells.

The concept that helminth-induced B cells can protect against allergic inflammation has been extended to other helminth infections that are natural for mice: in H polygyrus-infected mice, adoptive transfer of mesenteric lymph node B cells suppressed both DerP1-specific airway inflammation and EAE [55]. Interestingly, Breg cell development can also be seen during the infections caused by Leishmania major [56]; IL-10-producing B cells were critical for the development of unprotective TH2 responses and susceptibility to infection. In addition, murine cytomegalovirus has been shown to induce IL-10-producing Breg cells, resulting in decreased virus-specific CD81 T-cell responses and plasma cell expansion [57]. Other studies investigated the role that B cells play in infection with the nematode *Brugia pahangi*. Adoptively transferred peritoneal B cells, isolated from wild-type mice that had been immunized with *B. pahangi*, have been shown to protect athymic recipient mice against infection by *B. pahangi* infection [58].

In another study, the depletion of B cells from splenocytes of infected mice resulted in a reduced level of antigen-specific CD4^+^ T-cell proliferation paralleled by a reduced level of CD80 and CD86. Similar results were obtained if IL-10 was neutralized at the time of infection, suggesting that B cells producing IL-10 might modulate immune response in filarial-infected mice, via the suppression of CD80 and CD86 expression on Bregs [59]. Taken together, these studies show that helminths can induce Breg cells that can protect against allergic diseases via the release of IL-10 and that this process is particularly active during the chronic stage of infection. Furthermore, it can be concluded that the suppressive ability of Breg cells is not restricted to TH1 immune responses associated with autoimmunity, and the effect that the humoral immune response and FcR interactions might play a major role in controlling.

## 4. Bregs in Cancer

Given that Bregs have been shown to suppress autoimmunity via the inhibition of autoreactive T cells, it might be anticipated that Bregs could also downregulate the protective cytotoxic T lymphocyte responses directed against tumor cells. Terabe found that enhanced antitumor immunity can be seen in B-cell-deficient mice and is associated with an increased activity of T and NK cells, both of which are important for the promotion of natural tumor surveillance [60]. Increased CD4^+^ and CD8^+^ T-cell responses to TS/A tumors are observed in μMT mice [61]. In B16 melanoma, mature B-cell depletion using CD20 mAb dramatically exacerbates tumor progression and metastasis, arguing that B cells primarily support antitumor immune responses in this model [1]. Thus, these results suggest that B cells can also negatively regulate tumor immunity.

Scott and colleagues have shown that the interaction between CD40^L^ expressed on tumors and CD40 on B cells induces IL-10 production by B cells, indicating that the release of IL-10 is probably responsible for the diminished IFN*γ* production by CD8^+^ T and NK cells and the decreased CD8^+^ T-cell memory development. However, there is some evidence to suggest that IL-10 can suppress angiogenesis and, thus, encourage tumor regression [62] [63]. Furthermore, the ubiquitous expression of CD40 *in vivo* will probably lead to a more complex cascade of anti- and pro-inflammatory cytokines, which might overcome the inhibitory effect of Bregs. But it remains to be formally proven whether or not administration of anti-CD40 can generate Bregs *in vivo*. Another study demonstrated an excessively zealous Breg population may promote tumor cell growth, as one of the mechanisms used by tumor cells to escape from the immune response consists in activation of Bregs that produce TGF-beta [64].

A role for B cells in the development of tumor immunity has been assessed using μMT mice given Friend murine leukemia virus gag-expressing mouse EL-4, D5 melanoma, or MCA304 sarcoma cells. Inoue and colleagues have tested wild-type mice were unable to control tumor progression, whereas EL-4 gag and D5 tumors (but not MCA304) were eliminated in μMT mice, which developed tumor-specific cytotoxic T lymphocytes after tumor challenge. Similar study suggested the growth of EL4 thymoma, MC38 colon carcinoma, and B16 melanoma was prevented or slowed in μMT mice in contrast to control mice [62] [65].

By contrast, B-cell depletion using CD20 mAb in a syngeneic lymphoma model dramatically enhances tumor clearance through B10-cell elimination. Thus, as in autoimmunity, B10 cells are likely to be involved in regulating antitumor immunity. However, this regulation will be significantly influenced by the immunogenicity of the tumor and the nature of the antitumor immune response [66].

## 5. Summary

Despite the extensive efforts on the characterization of BREG subtypes and their mechanism of action in different animal models, questions still exist to unravel the mechanisms underlying Bregs biology and function. The precise phenotype and characteristic markers of Bregs are still the subject of debate. It remains unclear whether Bregs require self-reactive BCRs for function. Are Breg cells a developmentally distinct B-cell subset? Do Breg cells display a specific transcriptional signature such as FoxP3 for regulatory T cells? Understanding these questions may open novel avenues for the treatment of inflammatory diseases such as allergy and autoimmunity.

## References

[1] DiLillo DJ, Hamaguchi Y, Ueda Y, Yang K, Uchida J, Haas KM, Kelsoe G, Tedder TF (2008). Maintenance of Long-Lived Plasma Cells and Serological Memory despite Mature and Memory B Cell Depletion during CD20 Immunotherapy in Mice. Journal of Immunology.

[2] LeBien TW, Tedder TF (2008). B Lymphocytes: How They Develop and Function. Blood.

[3] Linton PJ, Harbertson J, Bradley LM (2000). A Critical Role for B Cells in the Development of Memory CD4 Cells. Journal of Immunology.

[4] Crawford A, Macleod M, Schumacher T, Corlett L, Gray D (2006). Primary T Cell Expansion and Differentiation *in Vivo* Requires Antigen Presentation by B Cells. Journal of Immunology.

[5] Bouaziz JD, Yanaba K, Venturi GM, Wang Y, Tisch RM, Poe JC, Tedder TF (2007). Therapeutic B Cell Depletion Impairs Adaptive and Autoreactive CD4+ T Cell Activation in Mice. Proceedings of the National Academy of Sciences of the United States of America.

[6] Homann D, Tishon A, Berger DP, Weigle WO, von Herrath MG, Oldstone MB (1998). Evidence for an Underlying CD4 Helper and CD8 T-Cell Defect in B-Cell-Deficient Mice: Failure to Clear Persistent Virus Infection after Adoptive Immunotherapy with Virus-Specific Memory Cells from muMT/muMT Mice. Journal of Virology.

[7] Bergmann CC, Ramakrishna C, Kornacki M, Stohlman SA (2001). Impaired T Cell Immunity in B Cell-Deficient Mice Following Viral Central Nervous System Infection. Journal of Immunology.

[8] O’Neill SK, Cao Y, Hamel KM, Doodes PD, Hutas G, Finnegan A (2007). Expression of CD80/86 on B Cells Is Essential for Autoreactive T Cell Activation and the Development of Arthritis. Journal of Immunology.

[9] Morris A, Moller G (1968). Regulation of Cellular Antibody Synthesis Effect of Adoptively Transferred Antibody-Producing Spleen Cells on Cellular Antibody Synthesis. Journal of Immunology.

[10] Shimamura T, Hashimoto K, Sasaki S (1982). Feedback Suppression of the Immune Response *in Vivo*. I. Immune B Cells Induce Antigen-Specific Suppressor T Cells. Cellular Immunology.

[11] L’Age-Stehr J, Teichmann H, Gershon RK, Cantor H (1980). Stimulation of Regulatory T Cell Circuits by Immunoglobulin-Dependent Structures on Activated B Cells. European Journal of Immunology.

[12] Shimamura T, Habu S, Hashimoto K, Sasaki S (1984). Feedback Suppression of the Immune Response *in Vivo*. III. Lyt-1^+^ B Cells Are Suppressor-Inducer Cells. Cellular Immunology.

[13] Kennedy MW, Thomas DB (1983). A Regulatory Role for the Memory B Cell as Suppressor-Inducer of Feedback Control. The Journal of Experimental Medicine.

[14] Fillatreau S, Sweenie CH, McGeachy MJ, Gray D, Anderton SM (2002). B Cells Regulate Autoimmunity by Provision of IL-10. Nature Immunology.

[15] Mizoguchi A, Mizoguchi E, Takedatsu H, Blumberg RS, Bhan AK (2002). Chronic Intestinal Inflammatory Condition Generates IL-10-Producing Regulatory B Cell Subset Characterized by CD1d Upregulation. Immunity.

[16] Mauri C, Gray D, Mushtaq N, Londei M (2003). Prevention of Arthritis by Interleukin 10-Producing B Cells. The Journal of Experimental Medicine.

[17] Yanaba K, Bouaziz JD, Haas KM, Poe JC, Fujimoto M, Tedder TF (2008). A Regulatory B Cell Subset with a Unique CD1d^hi^CD5^+^ Phenotype Controls T Cell-Dependent Inflammatory Responses. Immunity.

[18] Matsushita T, Yanaba K, Bouaziz JD, Fujimoto M, Tedder TF (2008). Regulatory B Cells Inhibit EAE Initiation in Mice While Other B Cells Promote Disease Progression. Journal of Clinical Investigation.

[19] Yanaba K, Bouaziz JD, Matsushita T, Tsubata T, Tedder TF (2009). The Development and Function of Regulatory B Cells Expressing IL-10 (B10 Cells) Requires Antigen Receptor Diversity and TLR Signals. The Journal of Immunology.

[20] Harris DP, Haynes L, Sayles PC, Duso DK, Eaton SM, Lepak NM, Johnson LL, Swain SL, Lund FE (2000). Reciprocal Regulation of Polarized Cytokine Production by Effector B and T Cells. Nature Immunology.

[21] Brummel R, Lenert P (2005). Activation of Marginal Zone B Cells from Lupus Mice with Type A(D) CpG-Oligodeoxynucleotides. The Journal of Immunology.

[22] Evans JG, Chavez-Rueda KA, Eddaoudi A, Meyer-Bahlburg A, Rawlings DJ, Ehrenstein MR, Mauri C (2007). Novel Suppressive Function of Transitional 2 B Cells in Experimental Arthritis. The Journal of Immunology.

[23] Gray M, Miles K, Salter D, Gray D, Savill J (2007). Apoptotic Cells Protect Mice from Autoimmune Inflammation by the Induction of Regulatory B Cells. Proceedings of the National Academy of Sciences of the United States of America.

[24] Burke F, Stagg AJ, Bedford PA, English N, Knight SC (2004). IL-10-Producing B220^+^CD11c^−^ APC in Mouse Spleen. The Journal of Immunology.

[25] Spencer NF, Daynes RA (1997). IL-12 Directly Stimulates Expression of IL-10 by CD5^+^ B Cells and IL-6 by Both CD5^+^ and CD5^−^ B Cells: Possible Involvement in Age-Associated Cytokine Dysregulation. International Immunology.

[26] O’Garra A, Stapleton G, Dhar V, Pearce M, Schumacher J, Rugo H, Barbis D, Stall A, Cupp J, Moore K (1990). Production of Cytokines by Mouse B Cells: B Lymphomas and Normal B Cells Produce Interleukin 10. International Immunology.

[27] Zhang X, Deriaud E, Jiao X, Braun D, Leclerc C, Lo-Man R (2007). Type I Interferons Protect Neonates from Acute Inflammation through Interleukin 10-Producing B Cells. The Journal of Experimental Medicine.

[28] Wolf SD, Dittel BN, Hardardottir F, Janeway CA (1996). Experimental Autoimmune Encephalomyelitis Induction in Genetically B Cell-Deficient Mice. The Journal of Experimental Medicine.

[29] Lampropoulou V, Hoehlig K, Roch T, Neves P, Calderon Gomez E, Sweenie CH, Hao Y, Freitas AA, Steinhoff U, Anderton SM, Fillatreau S (2008). TLR-Activated B Cells Suppress T Cell-Mediated Autoimmun-ity. The Journal of Immunology.

[30] Matsushita T, Fujimoto M, Hasegawa M, Komura K, Takehara K, Tedder TF, Sato S (2006). Inhibitory Role of CD19 in the Progression of Experimental Autoimmune Encephalomyelitis by Regulating Cytokine Response. American Journal of Pathology.

[31] Ray A, Basu S, Williams CB, Salzman NH, Dittel BN (2012). A Novel IL-10-Independent Regulatory Role for B Cells in Suppressing Autoimmunity by Maintenance of Regulatory T Cells via GITR Ligand. The Journal of Immunology.

[32] Hauser SL, Waubant E, Arnold DL, Vollmer T, Antel J, Fox RJ, Bar-Or A, Panzara M, Sarkar N, Agar-wal S, Langer-Gould A, Smith CH (2008). B-Cell Depletion with Rituximab in Relapsing-Remitting Multiple Sclerosis. The New England Journal of Medicine.

[33] Bar-Or A, Calabresi PA, Arnold D, Markowitz C, Shafer S, Kasper LH, Waubant E, Gazda S, Fox RJ, Panzara M, Sarkar N, Agarwal S, Smith CH (2008). Rituximab in Relapsing-Remitting Multiple Sclerosis: A 72-Week, Open-Label, Phase I Trial. Annals of Neurology.

[34] Anderson MS, Bluestone JA (2005). The NOD Mouse: A Model of Immune Dysregulation. Annual Review of Immunology.

[35] Xiu Y, Wong CP, Bouaziz JD, Hamaguchi Y, Wang Y, Pop SM, Tisch RM, Tedder TF (2008). B Lymphocyte Depletion by CD20 Monoclonal Antibody Prevents Diabetes in Nonobese Diabetic Mice Despite Iso-type-Specific Differences in Fc Gamma R Effector Functions. The Journal of Immunology.

[36] Hussain S, Delovitch TL (2007). Intravenous Transfusion of BCR-Activated B Cells Protects NOD Mice from Type 1 Diabetes in an IL-10-Dependent Manner. The Journal of Immunology.

[37] Tian J, Zekzer D, Hanssen L, Lu Y, Olcott A, Kaufman DL (2001). Lipopolysaccharide-Activated B Cells Down-Regulate Th1 Immunity and Prevent Autoimmune Diabetes in Nonobese Diabetic Mice. The Journal of Immunology.

[38] Trentham DE, Townes AS, Kang AH (1977). Autoimmunity to Type II Collagen an Experimental Model of Arthritis. The Journal of Experimental Medicine.

[39] Courtenay JS, Dallman MJ, Dayan AD, Martin A, Mosedale B (1980). Immunisation against Heterologous Type II Collagen Induces Arthritis in Mice. Nature.

[40] Wordsworth BP, Lanchbury JS, Sakkas LI, Welsh KI, Panayi GS, Bell JI (1989). HLA-DR4 Subtype Frequencies in Rheumatoid Arthritis Indicate That DRB1 Is the Major Susceptibility Locus within the HLA Class II Region. Proceedings of the National Academy of Sciences of the United States of America.

[41] Brunsberg U, Gustafsson K, Jansson L, Michaelsson E, Ahrlund-Richter L, Pettersson S, Mattsson R, Holmdahl R (1994). Expression of a Transgenic Class II Ab Gene Confers Susceptibility to Collagen-Induced Arthritis. European Journal of Immunology.

[42] Yanaba K, Hamaguchi Y, Venturi GM, Steeber DA, St Clair EW, Tedder TF (2007). B Cell Depletion Delays Collagen-Induced Arthritis in Mice: Arthritis Induction Requires Synergy between Humoral and Cell-Mediated Immunity. The Journal of Immunology.

[43] Gu Y, Yang J, Ouyang X, Liu W, Li H, Bromberg J, Chen SH, Mayer L, Unkeless JC, Xiong H (2008). Interleukin 10 Suppresses Th17 Cytokines Secreted by Macrophages and T Cells. European Journal of Immunology.

[44] Watanabe R, Ishiura N, Nakashima H, Kuwano Y, Okochi H, Tamaki K, Sato S, Tedder TF, Fujimoto M (2010). Regulatory B Cells (B10 Cells) Have a Suppressive Role in Murine Lupus: CD19 and B10 Cell Deficiency Exacerbates Systemic Autoimmunity. The Journal of Immunology.

[45] Haas KM, Watanabe R, Matsushita T, Nakashima H, Ishiura N, Okochi H, Fujimoto M, Tedder TF (2010). Protective and Pathogenic Roles for B Cells during Systemic Autoimmunity in NZB/W F1 Mice. The Journal of Immunology.

[46] Teichmann LL, Kashgarian M, Weaver CT, Roers A, Muller W, Shlomchik MJ (2011). B Cell-Derived IL-10 Does Not Regulate Spontaneous Systemic Autoimmunity in MRL.*Fas^lpr^* Mice. The Journal of Immunology.

[47] Mizoguchi A, Mizoguchi E, Smith RN, Preffer FI, Bhan AK (1997). Suppressive Role of B Cells in Chronic Colitis of T Cell Receptor Alpha Mutant Mice. The Journal of Experimental Medicine.

[48] Mizoguchi A, Bhan AK (2006). A Case for Regulatory B Cells. The Journal of Immunology.

[49] Yanaba K, Yoshizaki A, Asano Y, Kadono T, Tedder TF, Sato S (2011). IL-10-Producing Regulatory B10 Cells Inhibit Intestinal Injury in a Mouse Model. American Journal of Pathology.

[50] Wei B, Velazquez P, Turovskaya O, Spricher K, Aranda R, Kronenberg M, Birnbaumer L, Braun J (2005). Mesenteric B Cells Centrally Inhibit CD4^+^ T Cell Colitis through Interaction with Regulatory T Cell Subsets. Proceedings of the National Academy of Sciences of the United States of America.

[51] Amu S, Saunders SP, Kronenberg M, Mangan NE, Atzberger A, Fallon PG (2010). Regulatory B Cells Prevent and Reverse Allergic Airway Inflammation via FoxP3-Positive T Regulatory Cells in a Murine Model. Journal of Allergy and Clinical Immunology.

[52] Jankovic D, Cheever AW, Kullberg MC, Wynn TA, Yap G, Caspar P, Lewis FA, Clynes R, Ravetch JV, Sher A (1998). CD4^+^ T Cell-Mediated Granulomatous Pathology in Schistosomiasis Is Downregulated by a B Cell-Dependent Mechanism Requiring Fc Receptor Signaling. The Journal of Experimental Medicine.

[53] Mangan NE, Fallon RE, Smith P, van Rooijen N, McKenzie AN, Fallon PG (2004). Helminth Infection Protects Mice from Anaphylaxis via IL-10-Producing B Cells. The Journal of Immunology.

[54] Saaf AM, Halbleib JM, Chen X, Yuen ST, Leung SY, Nelson WJ, Brown PO (2007). Parallels between Global Transcriptional Programs of Polarizing Caco-2 Intestinal Epithelial Cells *in Vitro* and Gene Expression Programs in Normal Colon and Colon Cancer. Molecular Biology of the Cell.

[55] Wilson MS, Taylor MD, O’Gorman MT, Balic A, Barr TA, Filbey K, Anderton SM, Maizels RM (2010). Helminth-Induced CD19^+^CD23^hi^ B Cells Modulate Experimental Allergic and Autoimmune Inflammation. Eu-ropean Journal of Immunology.

[56] Ronet C, Hauyon-La Torre Y, Revaz-Breton M, Mastelic B, Tacchini-Cottier F, Louis J, Launois P (2009). Regulatory B Cells Shape the Development of Th2 Immune Responses in BALB/c Mice Infected with Leishmania Major through IL-10 Production. The Journal of Immunology.

[57] Madan R, Demircik F, Surianarayanan S, Allen JL, Divanovic S, Trompette A, Yogev N, Gu Y, Khodoun M, Hildeman D, Boespflug N, Fogolin MB, Grobe L, Greweling M, Finkelman FD, Cardin R, Mohrs M, Muller W, Waisman A, Roers A, Karp CL (2009). Nonredundant Roles for B Cell-Derived IL-10 in Immune Counter-Regulation. The Journal of Immunology.

[58] Paciorkowski N, Shultz LD, Rajan TV (2003). Primed Peritoneal B Lymphocytes Are Sufficient to Transfer Protection against *Brugia pahangi* Infection in Mice. Infection and Immunity.

[59] Gillan V, Lawrence RA, Devaney E (2005). B Cells Play a Regulatory Role in Mice Infected with the L3 of *Brugia pahangi*. International Immunology.

[60] Terabe M, Swann J, Ambrosino E, Sinha P, Takaku S, Hayakawa Y, Godfrey DI, Ostrand-Rosenberg S, Smyth MJ, Berzofsky JA (2005). A Nonclassical Non-V*α*14J*α*18 CD1d-Restricted (Type II) NKT Cell Is Sufficient for Down-Regulation of Tumor Immunosurveillance. The Journal of Experimental Medicine.

[61] Qin Z, Richter G, Schuler T, Ibe S, Cao X, Blankenstein T (1998). B Cells Inhibit Induction of T Cell-Dependent Tumor Immunity. Nature Medicine.

[62] Inoue S, Leitner WW, Golding B, Scott D (2006). Inhibitory Effects of B Cells on Antitumor Immunity. Cancer Research.

[63] Rowe V, Banovic T, MacDonald KP, Kuns R, Don AL, Morris ES, Burman AC, Bofinger HM, Clouston AD, Hill GR (2006). Host B Cells Produce IL-10 Following TBI and Attenuate Acute GVHD after Allogeneic Bone Marrow Transplantation. Blood.

[64] Olkhanud PB, Damdinsuren B, Bodogai M, Gress RE, Sen R, Wejksza K, Malchinkhuu E, Wersto RP, Biragyn A (2011). Tumor-Evoked Regulatory B Cells Promote Breast Cancer Metastasis by Converting Resting CD4^+^ T Cells to T-Regulatory Cells. Cancer Research.

[65] Shah S, Divekar AA, Hilchey SP, Cho HM, Newman CL, Shin SU, Nechustan H, Challita-Eid PM, Segal BM, Yi KH, Rosenblatt JD (2005). Increased Rejection of Primary Tumors in Mice Lacking B Cells: Inhibition of Anti-Tumor CTL and TH1 Cytokine Responses by B Cells. International Journal of Cancer.

[66] Minard-Colin V, Xiu Y, Poe JC, Horikawa M, Magro CM, Hamaguchi Y, Haas KM, Tedder TF (2008). Lymphoma Depletion during CD20 Immunotherapy in Mice Is Mediated by Macrophage Fc*γ*RI, Fc*γ*RIII, and Fc*γ*RIV. Blood.

